# Lifespan benefits for the combination of rapamycin plus acarbose and for captopril in genetically heterogeneous mice

**DOI:** 10.1111/acel.13724

**Published:** 2022-09-30

**Authors:** Randy Strong, Richard A. Miller, Catherine J. Cheng, James F. Nelson, Jonathan Gelfond, Shailaja Kesaraju Allani, Vivian Diaz, Angela Olsen Dorigatti, Jonathan Dorigatti, Elizabeth Fernandez, Andrzej Galecki, Brett Ginsburg, Karyn L. Hamilton, Martin A. Javors, Kerry Kornfeld, Matt Kaeberlein, Suja Kumar, David B. Lombard, Marisa Lopez‐Cruzan, Benjamin F. Miller, Peter Rabinovitch, Peter Reifsnyder, Nadia A. Rosenthal, Molly A. Bogue, Adam B. Salmon, Yousin Suh, Eric Verdin, Herbert Weissbach, John Newman, Francesca Maccchiarini, David E. Harrison

**Affiliations:** ^1^ Geriatric Research, Education and Clinical Center and Research Service, South Texas Veterans Health Care System, Department of Pharmacology Barshop Institute for Longevity and Aging Studies at The University of Texas Health Science Center at San Antonio Texas USA; ^2^ Department of Pathology and Geriatrics Center University of Michigan Ann Arbor Michigan USA; ^3^ Department of Cellular and Integrative Physiology Barshop Institute for Longevity and Aging Studies at The University of Texas Health Science Center at San Antonio Texas USA; ^4^ Department of Population Health Sciences University of Texas Health Science Center at San Antonio Texas USA; ^5^ Florida Atlantic University Jupiter Florida USA; ^6^ Geriatric Research, Education and Clinical Center, South Texas Veterans Health Care System, Department of Molecular Medicine Barshop Institute for Longevity and Aging Studies at The University of Texas Health Science Center at San Antonio Texas USA; ^7^ Departments of Internal Medicine and Biostatistics University of Michigan School of Medicine and School of Public Health Ann Arbor Michigan USA; ^8^ Department of Psychiatry University of Texas Health Science Center at San Antonio Texas USA; ^9^ Department of Health and Exercise Science and the Center for Healthy Aging Colorado State University Fort Collins Colorado USA; ^10^ Department of Developmental Biology Washington University School of Medicine St. Louis Missouri USA; ^11^ Department of Laboratory Medicine and Pathology University of Washington Seattle Washington USA; ^12^ Department of Internal Medicine University of Michigan Ann Arbor Michigan USA; ^13^ Aging and Metabolism Research Program, Oklahoma Medical Research Foundation (OMRF), Oklahoma Nathan Shock Center, Oklahoma Center for Geroscience Harold Hamm Diabetes Center Oklahoma City Oklahoma USA; ^14^ The Jackson Laboratory Bar Harbor Maine USA; ^15^ Department of Obstetrics & Gynecology, Department of Genetics & Development, Reproductive Aging Program Vagelos College of Physicians & Surgeons Columbia University New York New York USA; ^16^ Buck Institute for Research on Aging Novato California USA; ^17^ Division of Geriatrics University of California San Francisco California USA; ^18^ Division of Aging Biology National Institute on Aging Bethesda Maryland USA

**Keywords:** acarbose plus rapamycin, captopril, survival

## Abstract

Mice bred in 2017 and entered into the C2017 cohort were tested for possible lifespan benefits of (R/S)‐1,3‐butanediol (BD), captopril (Capt), leucine (Leu), the Nrf2‐activating botanical mixture PB125, sulindac, syringaresinol, or the combination of rapamycin and acarbose started at 9 or 16 months of age (RaAc9, RaAc16). In male mice, the combination of Rapa and Aca started at 9 months and led to a longer lifespan than in either of the two prior cohorts of mice treated with Rapa only, suggesting that this drug combination was more potent than either of its components used alone. In females, lifespan in mice receiving both drugs was neither higher nor lower than that seen previously in Rapa only, perhaps reflecting the limited survival benefits seen in prior cohorts of females receiving Aca alone. Capt led to a significant, though small (4% or 5%), increase in female lifespan. Capt also showed some possible benefits in male mice, but the interpretation was complicated by the unusually low survival of controls at one of the three test sites. BD seemed to produce a small (2%) increase in females, but only if the analysis included data from the site with unusually short‐lived controls. None of the other 4 tested agents led to any lifespan benefit. The C2017 ITP dataset shows that combinations of anti‐aging drugs may have effects that surpass the benefits produced by either drug used alone, and that additional studies of captopril, over a wider range of doses, are likely to be rewarding.

## Graphical abstract

The C2017 ITP dataset shows that combinations of anti‐aging drugs may have effects that surpass the benefits produced by either drug used alone. The data further show that males benefit more than females from the combination of RAPA and acarbose.

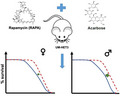

**Abbreviations**
17αE2: 17‐α‐estradiol
ACA: Acarbose
ACE: Angiotensin converting enzyme
ANOVA: Analysis of Variance
BCAA: Branched chain amino acid.
BD: 1,3‐butanediol
Capt: Captopril
ITP: Intervention Testing Program
IVC: Individually ventilated cages
Leu: Leucine
NDGA: Nordihydroguaiaretic acid
RaAc16: Rapamycin plus acarbose, starting at 16 months of age
RaAc9: Rapamycin plus acarbose, starting at 9 months of age
RAPA: Rapamycin
Sul: Sulindac
Syr: Syringaresinol
TJL: The Jackson Laboratory
UM: University of Michigan
UT: University of Texas Health Science Center at San Antonio

## 1 INTRODUCTION

The National Institute on Aging Interventions Testing Program (ITP) evaluates agents hypothesized to extend lifespan, presumably by altering fundamental processes of aging or attenuating/delaying age‐related diseases. The goals of the ITP are (1) to gain insight into mechanisms of aging, (2) to provide a platform for preclinical assessment of potential life‐ and health‐span enhancing therapeutics, and (3) to examine the efficacy and test for potential deleterious effects of agents already offered to the public (Nadon et al., 2008). The proposals for test agents are solicited annually from the research community, screened by two levels of review, and chosen based on the strength of the evidence that the agents may extend the life and slow aging (Nadon et al., 2008). Notable strengths of the program are the rigor and reproducibility of its design and the genetic heterogeneity of the animal model. All agents are tested in parallel at three sites: The Jackson Laboratory (TJL); the University of Michigan (UM); and the University of Texas Health Science Center at San Antonio (UT). Standard operating procedures, including diet formulation and distribution, animal breeding, husbandry, and monitoring criteria, are replicated across sites (Nadon et al., 2008). Agents are tested in genetically heterogeneous (UM‐HET3) mice to minimize strain‐specific effects. The effects of all agents on survival and other age‐sensitive traits are published.

Eight of the 35 agents tested by the ITP have significantly increased lifespan in one or both sexes. None has shortened lifespan. Five of the 8, that is, nordihydroguaiaretic acid (NDGA, Strong et al., 2008; Harrison et al., 2014; Strong et al., 2016), aspirin (Strong et al., 2008), 17‐α‐estradiol (17⟨E2) (Harrison et al., 2014; Harrison et al., 2021; Strong et al., 2016), Protandim (Strong et al., 2016), and canagliflozin (Miller et al., 2020) have increased lifespan only in males. None of the agents tested to date has increased lifespan only in females. Three agents led to significant lifespan increased in both sexes, but with varying degrees of sex‐specificity. Glycine, for example, led to small but similar increases, significant in both sexes (Miller et al., 2019). Acarbose effects were more dramatic in males than in females at any of the three tested doses (Harrison et al., 2019), and if started later in life, that is, at 20 months of age (Harrison et al., 2014; Strong et al., 2016; Harrison et al., 2019). Rapamycin, over a range of doses and at two starting ages, has had strong positive effects in both sexes (Harrison et al., 2009; Miller et al., 2011; Miller et al., 2014), but at a given dose in chow typically leads to a larger percentage increase in female than in male mice (Harrison et al., 2021). Interpretation of this sex‐specific effect is, however, complicated by data showing that blood levels of rapamycin are higher in female mice than in males given the same dose in chow, and it is unclear whether males and females with similar blood rapamycin levels would have different survival curves. Five of the agents that increase lifespan (NDGA, aspirin, rapamycin, 17⟨E2, and acarbose) have been re‐examined in later cohorts with different dosages and treatment durations. Of these, only aspirin did not replicate, although it was only tested at higher doses (Miller et al., 2019).

The ITP has also begun to test combinations of lifespan‐extending agents for potential additive effects. Metformin and rapamycin were tested in combination based on the hypothesis that the insulin‐sensitizing action of metformin might compensate for the potentially deleterious insulin desensitizing effect of rapamycin and potentially different mechanisms of action of the two agents might have additive effects on survival (Strong et al., 2016). The results were not entirely clear‐cut because in both sexes the combination of metformin and rapamycin led to a larger percentage benefit compared with simultaneous controls than had been seen in previous studies of rapamycin alone, but the benefit of the combination over rapamycin‐only historical controls was not statistically significant. Here, we extend this strategy, testing rapamycin and acarbose in combination, starting at 9 or 16 months of age, based on a similar rationale to that used for the metformin/rapamycin trial.

We further report the results of six drugs not previously tested. The rationales for testing these agents were as follows: The ketogenic effect of dietary (R/S)‐1,3‐butanediol was a major reason for its selection. Ketogenic diets, as well as β‐hydroxybutyrate, a major metabolic product of ketogenic diets, have been reported to increase survival and attenuate pathological as well as other age‐associated traits, and ameliorate aging phenotypes of the brain and heart (Newman et al., 2017; Roberts et al., 2017). Captopril, an FDA‐approved angiotensin‐converting enzyme (ACE) inhibitor used for the treatment of hypertension, was selected because it was reported to increase lifespan in both wildtype C. elegans and mutants that extend lifespan (Kumar et al., 2016). In addition, other ACE inhibitors are reported to extend lifespan in mice (Feder et al., 1993) and rats (Santos et al., 2009) as well as delay age‐related changes in the structure and function of the kidney, cardiovascular system, liver, and brain. L‐Leucine, a branched‐chain amino acid (BCAA) that bypasses liver catabolism after ingestion, thus reaching tissues intact, was chosen because it increased chronological lifespan in yeast (Alvers et al., 2009) and lengthened life in C. elegans (Mansfeld et al., 2015) and mice (D'Antona et al., 2010), either alone or in combination with other BCAAs. Leucine alone or in combination with other BCAA's is also reported to increase insulin sensitivity (Solerte et al., 2008) and muscle protein synthesis (Dickinson et al., 2014) in older humans. PB125, a formulation consisting of rosemary extract, ashwagandha extract, and luteolin, was selected because it was reported to produce greater activation of nuclear factor erythroid 2‐related factor 2 (Nrf2) (Hamilton, K. and Miller, B.F., unpublished data) than Protandim, previously reported by the ITP to extend median lifespan in male UMHET3 mice (Strong et al., 2016). Sulindac is an FDA‐approved non‐steroidal anti‐inflammatory drug (NSAID). The reason sulindac was selected is due to its ability to initiate a preconditioning response that protects against oxidative damage. More recently, it has been shown to protect against cardiac and brain ischemia–reperfusion damage (Moench et al., 2009, Modi et al., 2014), retinal epithelial cell degeneration (Sur et al., 2014) as well as promote toxicity to cancer cells (Marchetti et al., 2009; Ayyanathan et al., 2012). Syringaresinol, a specific component of the ginseng berry, has been shown to extend the lifespan of C. elegans and Drosophila and appears to act through FOXO3/sirtuin‐dependent mechanisms in C. elegans and inhibition of IGF1 signaling in mammals (Kim et al., 2020).

## 2 RESULTS

### 2.1 Standard analysis

The ITP has, since its inception in 2004, used a standard analytical protocol, in which each tested drug is compared, by a site‐stratified log‐rank test, to same‐sex controls, using data pooled from all three test sites. The reports include summary statistics, that is, median and 90th percentile age, from the pooled‐site data for each drug for each sex. The Wang/Allison statistic (Wang et al., 2004), a Fisher exact test on proportions surviving at the 90th percentile, is used as an index for a drug's potential to support exceptionally long survival. Each report also shows the corresponding statistics and p‐values for each site considered separately; even though these tests have much less statistical power than the pooled‐site data, they do provide useful insights into possible site‐specific heterogeneity in drug effects. We first present the results of this standard analysis, using data pooled from all three sites, before turning to an ad hoc analysis modified to adjust for the unusual features of the C2017 data set (i.e., the data set from the mice bred for this Cohort of mice).

**FIGURE 1:**
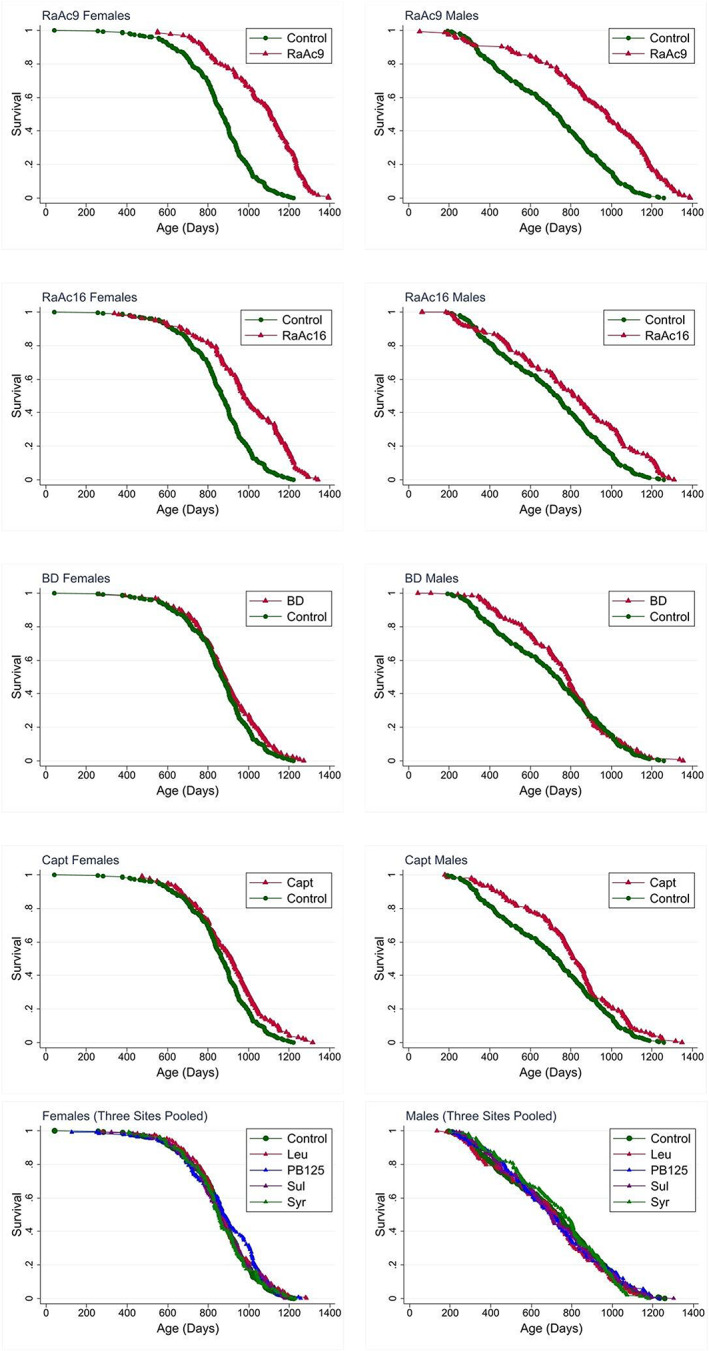
Survival plots for data pooled from all three sites:

*Rapamycin plus acarbose, starting at 9 months of age (RaAc9)*. This combination of agents produced a 28% increase in median lifespan in females (*p* < 0.0001) and a 34% increase in males (*p* < 0.0001) for data pooled across sites (Figure 1). This was the only treatment that significantly increased survival in both sexes at all three sites tested separately. In females, RaAc9 extended the median lifespan by 32% at TJL, 8% at UM, and 35% at UT. Table 1 presents the median, log‐rank p‐value (stratified by site), and 90th percentile age for each drug and each sex, with change scores, compared with sex‐matched control mice, along with the site‐stratified Wang/Allison p‐value, for data pooled across all three sites. Tables 2 and 3 shows similar statistics for each site presented separately. In males, RaAc9 extended a median lifespan of 13% at TJL, 25% at UM, and 49% at UT. RaAc9 also increased 90th percentile survival, for pooled data and at each site individually, both in females and males; the Wang/Allison p‐values are included in Tables 1, 2, 3.

**TABLE 1:** Life table statistics for mice pooled across all three test sites

Group	Count	Median	% Change Median	Log‐rank	p90	% Change p90	Wang‐Allison
Females							
Cont_17	276	868			1060		
BD	134	881	1	0.04	1101	4	0.16
Capt	132	915	5	0.002	1144	8	0.02
Leu	135	853	−2	0.20	1109	5	0.17
PB125	128	875	1	0.16	1094	3	0.48
RaAc16	134	976	12	0.001	1223	15	0.001
RaAc9	134	1107	28	0.001	1278	21	0.001
Sul	136	853	‐2	0.87	1081	2	0.30
Syr	136	853	‐2	0.99	1051	−1	1.00
Males							
Cont_17	285	718			1029		
BD	145	785	9	0.11	1066	4	0.40
Capt	150	821	14	0.001	1097	7	0.01
Leu	147	720	0	0.27	1020	−1	0.62
PB125	144	699	−3	0.64	1053	2	0.24
RaAc16	138	815	14	0.001	1217	18	0.001
RaAc9	153	981	37	0.001	1272	24	0.001
Sul	144	698	−3	0.69	1037	1	0.87
Syr	152	763	6	0.94	1011	−2	0.51

*Note*: p90 is 90th percentile age. Count, median, p90, and Wang/Allison calculations exclude mice removed for fighting and other causes, for which the endpoint (death or euthanasia when moribund) did not occur. Log‐rank test includes all mice entered into the study. Medians and p90 are shown in days. Percent change for median and p90 is calculated with respect to the median and p90 values for the pooled controls; they are not calculated as the mean value of site‐specific median percent changes. p‐values are not adjusted for multiple comparisons. Entries where *p* < 0.001 are tabulated at *p* = 0.001.

**TABLE 2:** Survival statistics for data from individual test sites (Females)

		Median	% Change Median	Log‐rank	p90	% Change p90	Wang‐Allison
Females at TJL							
Control	96	871			1068		
BD	48	929	7	0.04	1101	3	0.26
Capt	48	960	10	0.0032	1245	17	0.007
Leu	48	882	1	0.67	1109	4	0.99
PB125	48	895	3	0.34	1097	3	0.57
RaAc16	48	1052	21	0.001	1275	19	0.001
RaAc9	48	1147	32	0.001	1304	22	0.001
Sul	48	875	0	0.36	1127	6	0.09
Syr	48	896	3	0.29	1085	2	0.57
Females at UM							
Control	90	902			1094		
BD	43	826	−8	0.42	1101	1	0.77
Capt	44	890	−1	0.93	1086	−1	0.99
Leu	43	841	−7	0.49	1086	−1	0.99
PB125	44	885	−2	0.26	1056	−3	0.38
RaAc16	43	943	5	0.0131	1229	12	0.01
RaAc9	44	972	8	0.0003	1221	12	0.001
Sul	44	841	−7	0.03	1072	−2	0.55
Syr	44	831	−8	0.02	1046	−4	0.04
Females at UT							
Control	90	824			979		
BD	43	864	5	0.03	1129	15	0.14
Capt	40	872	6	0.02	1053	8	0.11
Leu	44	863	5	0.02	1112	14	0.07
PB125	36	869	5	0.01	1124	15	0.009
RaAc16	43	976	18	0.001	1209	23	0.001
RaAc9	42	1130	37	0.001	1250	28	0.001
Sul	44	850	3	0.19	1061	8	0.23
Syr	44	855	4	0.36	1087	11	0.77

**TABLE 3:** Survival Statistics for Data from Individual Test Sites (Males)

		Median	% Change Median	Log‐rank	p90	% Change p90	Wang‐Allison
Males at TJL							
Control	102	748			1079		
BD	54	764	2	0.85	1083	0	0.79
Capt	54	827	11	0.24	1081	0	0.79
Leu	51	718	−4	0.43	1034	−4	0.27
PB125	51	609	−19	0.42	1053	−2	0.58
RaAc16	54	739	−1	0.16	1201	11	0.09
RaAc9	54	847	13	0.001	1247	16	0.001
Sul	48	710	−5	0.57	1120	4	0.25
Syr	53	769	3	0.18	1018	−6	0.09
Males at UM							
Control	88	790			1019		
BD	42	753	−5	0.93	985	−3	0.99
Capt	45	744	−6	0.70	1099	8	0.23
Leu	45	720	−9	0.11	980	−4	0.77
PB125	42	731	−7	0.99	1053	3	0.35
RaAc16	33	809	2	0.08	1226	20	0.04
RaAc9	48	987	25	0.001	1274	25	0.001
Sul	45	696	−12	0.93	1067	5	0.55
Syr	48	743	−6	0.39	1007	−1	0.77
Males at UT							
Control	95	676			963		
BD	49	810	20	0.01	1066	11	0.15
Capt	51	866	28	0.001	1125	17	0.01
Leu	51	723	7	0.71	979	2	0.78
PB125	51	735	9	0.12	1053	9	0.15
RaAc16	51	897	33	0.001	1195	24	0.01
RaAc9	51	1014	50	0.001	1277	33	0.001
Sul	51	678	0	0.98	969	1	0.78
Syr	51	805	19	0.03	1000	4	0.39

*Rapamycin plus acarbose, starting at 16 months of age (RaAc16)*, increased median lifespan by 13% both in females (*p* < 0.0001) and in males (*p* < 0.0001), using data pooled across sites (Figure 1). Site‐specific analyses, shown in Tables 2 and 3, revealed a significant extension of lifespan at all sites for females, but only at UT for males. In females, RaAc16 extended median lifespan by 21% at TJL (*p* < 0.0001), 5% at UM (*p* = 0.01) and 18% at UT (*p* < 0.0001). RaAc16 increased 90th percentile survival for pooled data in both sexes (*p* < 0.001; Table 1) and at each site individually for females. In males, RaAc16 extended median lifespan of UT males by 32% (*p* < 0.0001). The RaAc16 effect on male survival was significant only at UT (Tables 2 and 3).

*Captopril, started at 5 months*, increased the median lifespan in females by 6% (*p* = 0.002) and in males by 13% (*p* = 0.001) when data were pooled across all three sites (Table 1). Single‐site analyses (Tables 2 and 3) showed a significant log‐rank statistic for females at both TJL (10%, *p* = 0.003) and UT (7%, *p* = 0.02), and for males at UT only (27%, *p* < 0.001). The Wang/Allison test implied a benefit of captopril on survival to the 90th percentile with *p* = 0.02 in females and *p* = 0.01 in males.

*1,3‐Butanediol*, Treatment with 1,3‐butanediol increased median lifespan of females by 2% (*p* = 0.04) for data pooled across all three sites (Table 1), with significant site‐specific effects noted at TJL (7%; *p* = 0.04) and UT (4%, *p* = 0.03). 1,3‐butanediol did not alter the survival of males.

*Leucine, PB125, sulindac, and syringaresinol*, did not produce significant effects on lifespan for data pooled across the three sites (Table 1). The site‐specific analyses (Tables 2 and 3) for these four agents suggested benefits for leucine and PB125 in females at UT and syringaresinol in males at UT, but none of the agents led to improved survival in either sex at TJL or UM. Figures S1–S5 present survival curves for butanediol (BD), RaAc9, RaAc16, Captopril, and the remaining four tested agents shown separately for each site and each sex.

### 2.2 Results of ad hoc analysis, omitting UT mice

The C2017 cohort presented an unusual feature, in that male and female untreated controls at UT were unusually short‐lived compared to controls in all previous UT cohorts. Table S1 shows survival for control males and females in all prior ITP cohorts. Considering mice born between 2004 and 2016, median survival for females has been remarkably consistent, both from year to year and from site to site, with means and coefficients of variance ranging between 2% and 3% at each of the three sites. Median survival for males, in contrast, has shown greater year‐to‐year variation (coefficients of variance ranging from 5% to 7% at the three sites), and in addition, has shown consistently higher median survival of males at UM, (mean difference of 90 days, *p* < 10^−4^), compared with males at the other two sites.

**FIGURE 2:**
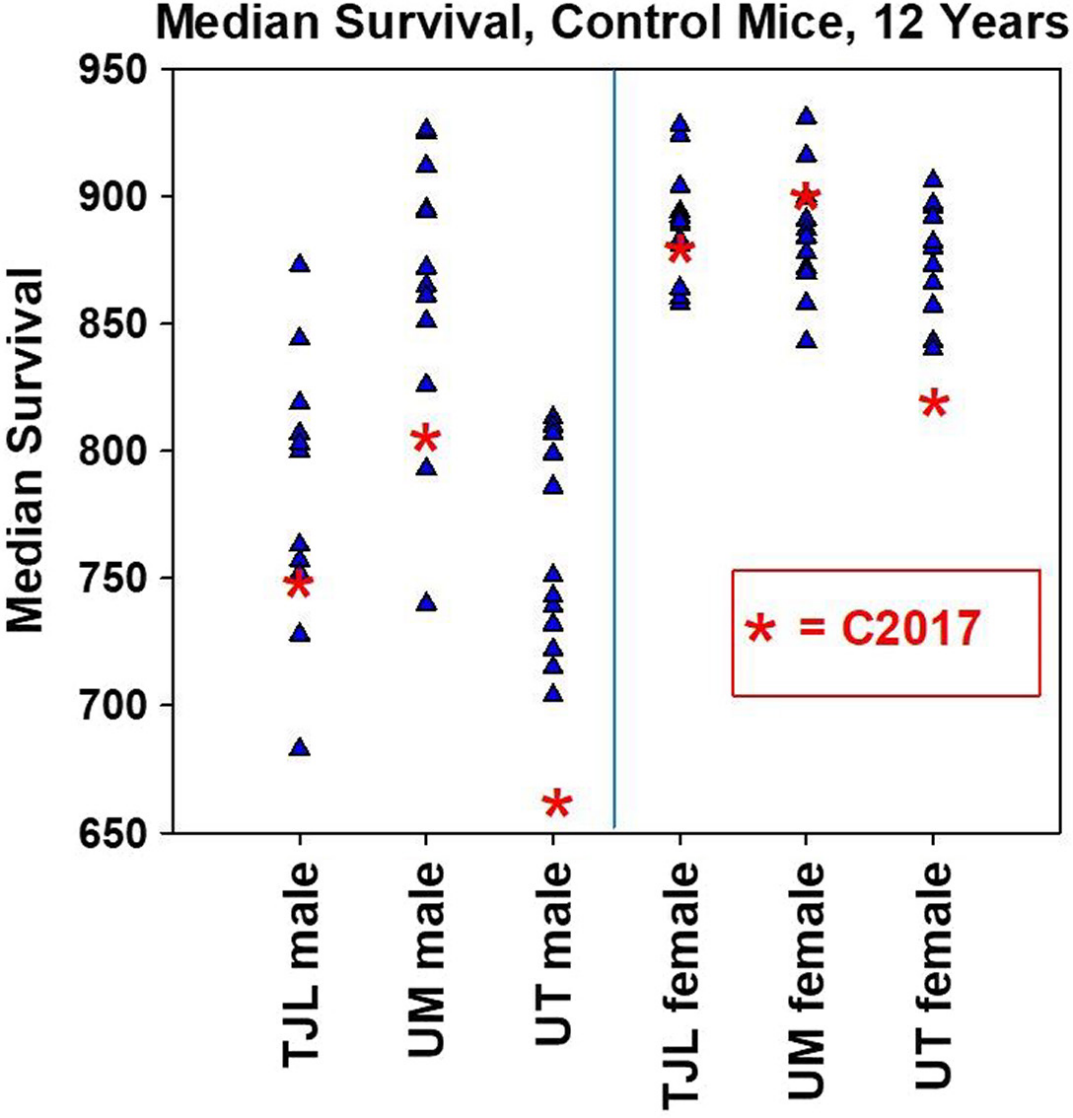
Median Survival for Controls at Each ITP Site, 2004–2017

Figure 2 shows dot plots comparing, for each site, the median survival of C2017 controls to the survival of control mice in C2017. The median lifespan of UT males in C2017, compared with the assumed‐normal distribution of UT males in prior cohorts, has a two‐tailed z‐score of 0.022; in other words, on average only one cohort in 45 would be expected to deviate from the average UT male control cohort, in either direction, by the amount seen in 2017. The corresponding one‐tailed z‐score of 0.011 implies that only one cohort in 90 would be expected to be as far below the average as the C2017 males at UT. Similarly, for females, only one cohort in 23 would be expected to deviate, in either direction, as far as the UT C2017 females, and only one cohort in 45 would be expected to be lower than average to the extent seen in the 2017 mice. The null hypothesis is that the C2017 males and C2017 females are each randomly and independently sampled from a notional collection of annual control cohorts of UT mice.

We considered the possibility that all C2017 mice—controls and drug‐treated—at UT might have been exposed to some unknown environmental factor that lowered median survival. We considered the six drugs not previously known to extend mouse lifespan and showed there are at each site 12 log‐rank tests available for comparison (6 drugs × 2 sexes) at the sites. The site‐specific analysis led to significant results for 6 of 12 such drug/sex analyses at UT, 2 of 12 at TJL, and 0 of 12 at UM. The null hypothesis here is that the distribution of beneficial results should not differ among test sites, and this hypothesis fails at *p* = 0.014 (Fisher's Exact test) for the C2017 data sets. The most parsimonious explanation for this set of results—both the unusually low survival of UT male and female controls, and the surprisingly high proportion of significant site‐specific benefits at UT—is that some unknown environmental factor may have impaired the survival of control mice, but not drug‐treated mice, at UT in C2017. If this is indeed the case, then the C2017 dataset violates one of the assumptions of our standard analysis, that is, that drug‐treated and control mice differ only with respect to the presence or absence of drug added to the chow. Pooling data across three sites, even one of which violates this assumption of our analytical approach, would produce invalid results and misleading inferences.

**FIGURE 3:**
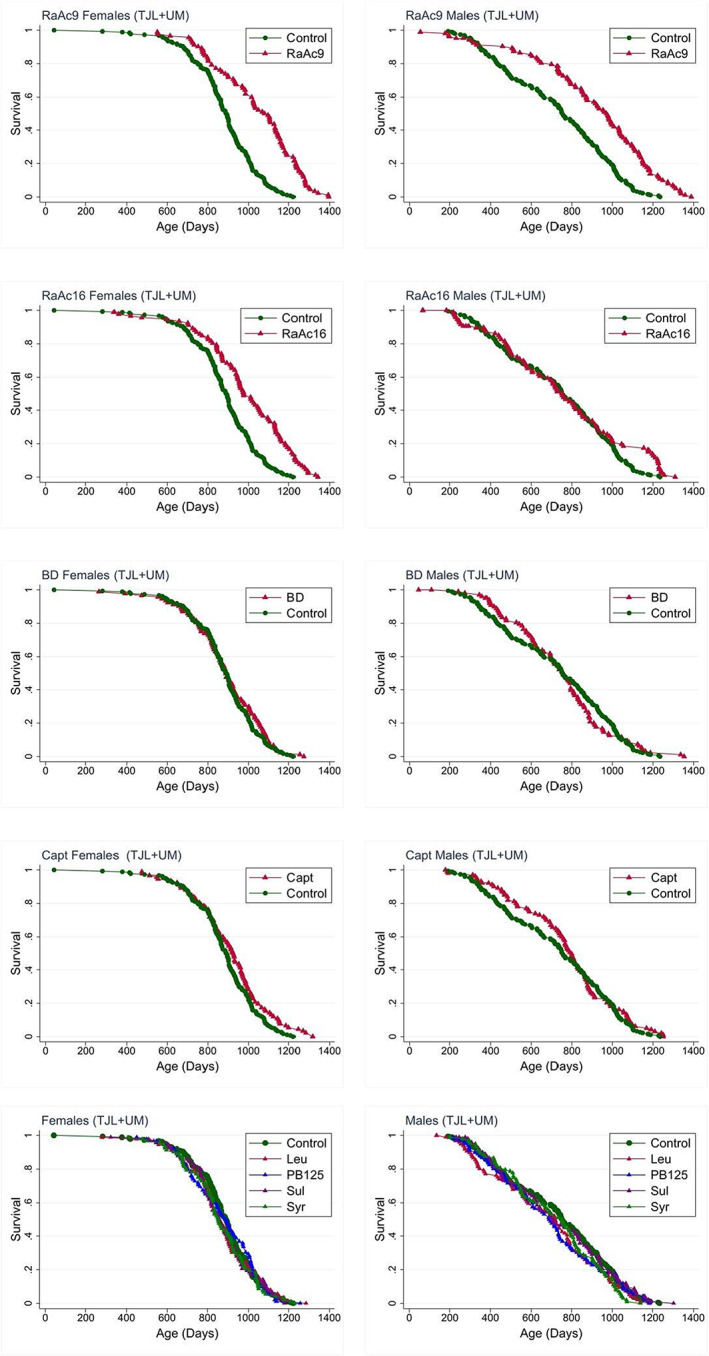
Survival plots for data pooled from TJL and UM sites

For that reason, we supplemented our standard three‐site pooled analysis with an additional set of calculations using the TJL and UM data only. Table 4 collects medians, log‐rank p‐values, and 90th percentile values with Wang/Allison p‐values, for the combined data from TJL and UM, omitting the UT mice. Survival curves for mice of each sex are collected in Figure 3.

**TABLE 4:** Survival Statistics Pooled from TJL and UM Sites Only

Group	*N*	Median	%Change Median	Log‐rank	p90	%Change p90	Wang‐Allison
Females							
Cont_17	186	890			1079		
BD	91	888	0	0.31	1101	2	0.15
Capt	92	925	4	0.04	1151	7	0.06
Leu	91	853	−4	0.88	1086	1	0.52
PB125	92	891	0	0.97	1056	−2	0.99
RaAc16	91	976	10	0.001	1231	14	0.001
RaAc9	92	1089	22	0.001	1278	18	0.001
Sul	92	856	−4	0.45	1088	1	0.53
Syr	92	852	−4	0.51	1049	−3	0.40
Males							
Cont_17	190	752			1046		
BD	96	756	0	0.94	1074	3	0.99
Capt	99	798	6	0.26	1085	4	0.22
Leu	96	719	−4	0.10	1034	−1	0.84
PB125	93	688	−9	0.55	1053	1	0.68
RaAc16	87	750	0	0.03	1223	17	0.004
RaAc9	102	973	29	0.001	1247	19	0.001
Sul	93	708	−6	0.64	1074	3	0.41
Syr	101	752	0	0.12	1011	−3	0.22

*Note*: See notes in Table 1.

*For RaAc9*, median increases for the (TJL + UM) data are 22% for females and 29% for males, similar to, though smaller than, the increases seen in the standard three‐site analysis, where the short‐lived UT controls were included in the comparison group. The 90th percentile ages were 18% and 19% above the control levels, both highly significant using the Wang/Allison method. For RaAc16, the log‐rank test was significant for both sexes, but the medians for males were the same as the medians for control males. The plots in Figure 3 show that the RaAc16 males differed from the control survival patterns only after 1000 days. The 90th percentile ages of RaAc16 mice were 14% higher for females and 17% higher for males. Thus, the data from the two‐site analysis lead to the same conclusion as for the standard three‐site analysis, though point estimates for change in the median and 90th percentile age are smaller.

*The two‐site analysis for Captopril* differs in some respects from the results of the standard analysis. For female mice, both datasets show a significant benefit in females, with increases of 4%–5% and significant log‐rank *p*‐values (0.04 and 0.002, respectively). The 90th percentile increases are 7%–8%, with similar p‐values for the Wang/Allison test (0.02 and 0.06, respectively, consistent with the lower power of the two‐site data). The effect of captopril on male mice in the two‐site dataset is not significant, showing a 6% change (*p* < 0.26), in contrast to the three‐site analysis where captopril led to a 14% increase at *p* = 0.002. Inspection of the survival curves for the TJL + UM data (Figure 3) shows that most of the benefit in female mice was observed in animals older than 800 days, while for males most of the survival benefit occurred in mice younger than 800 days.

*BD (1,3 butanediol)* did not lead to any increase in lifespan in the TJL + UM data set, with 0% change in the median and <3% change in the 90th percentile age. This is somewhat in contrast to the three‐site analysis, where BD effects on females reached significance at *p* = 0.04 despite a very small (2%) increase in the median. The results for leucine, PB125, sulindac and syringaresinol were qualitatively similar to those seen for the standard three‐site analysis: none of these four agents produced a significant effect by log‐rank or Wang/Allison statistic in either sex.

**FIGURE 4:**
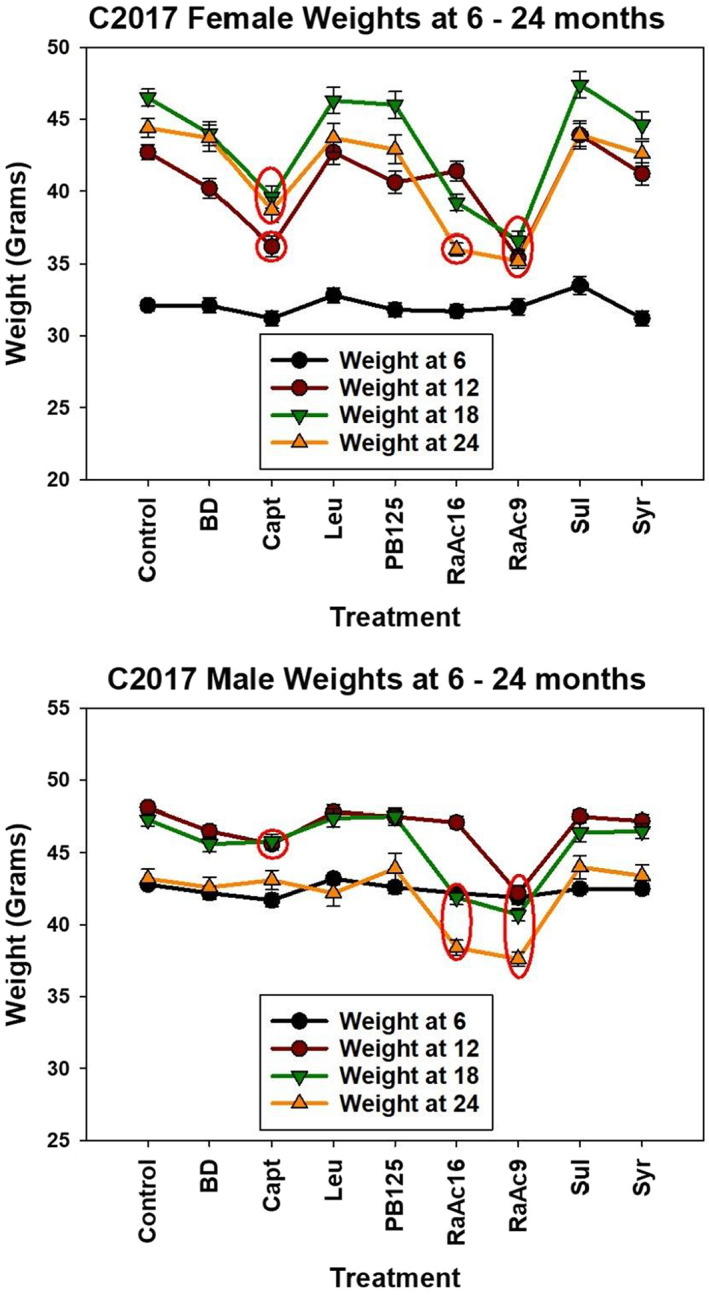
Weights at 6–24 months for C2017 groups (TJL, UM, UT Pooled). Each symbol shows mean and SEM for weight, in grams, for the indicated group of mice, pooled over all three sites. Females are shown in the top panel and males at the bottom. Red ovals indicate groups for which mice in the drug‐treated group differed at *p* < 0.05 from age‐ and sex‐matched control mice by Tukey's postdoc test after one‐factor ANOVA.

*Body weights* at ages 6–24 months are shown in Figure 4 for each combination of sex and treatment group. In both sexes, RaAc9 leads to lower body weight (i.e., less weight gain) than seen in controls at the ages of 12, 18, and 24 months. RaAc16 produces a significantly lower body weight, at 18 and 24 months in male mice and 24 months in females. Captopril‐treated females are lower in weight at 12, 18, and 24 months, and in males at 12 and 18 but not 24 months.

## 3 DISCUSSION

It is a well‐accepted principle of experimental science that analytical plans should be formulated prior to data acquisition and adhered to once data have been obtained since ad hoc modification of analysis strategy can lead to misinterpretations. The ITP has followed this principle since its inception in 2004. Occasionally, however, an unanticipated circumstance makes it necessary to consider supplementary analytical approaches, with a frank acknowledgment that conclusions drawn from this improvised approach do not have the strength of evidence that would emerge from cohorts that had gone through the experiment without incident. In the 2017 cohort, we noted such a circumstance—the unusually short survival times of both control males and controls females at UT—that prompted us to analyze our data twice, once using our standard method pooling across all three sites, and then again using a dataset from which the UT mice had been removed. We have presented both sets of statistics since we believe that some readers will find the first approach to be more reliable, and others will find the second approach more credible. In our view, each set of conclusions ought to be considered tentative, that is, less well supported than those that emerged from previous ITP publications, when they give different results, but well supported when the approaches agree.

Testing the combination of Rapa and Aca allowed us to test varying interpretations of the sex‐specific effects of Aca on survival. In past publications (Harrison et al., 2014; Harrison et al., 2019), Aca has produced significant lifespan increases in both sexes, but a greater percentage benefit in males than in females. We considered two possible classes of explanation for this: (I) that Aca produced benefits through interaction with a metabolic or physiological pathway particularly relevant to male longevity; or (II) that Aca produced equivalent benefits in both sexes, but also produced negative side effects with longevity effects in females only. Rapa, in contrast, produces strong lifespan benefits in both sexes, with higher percent changes in females consistent with the higher blood levels of Rapa in female mice than in males. If model I were correct, we would expect that the combination of Rapa and Aca would be beneficial in both sexes, equaling or surpassing historical results in mice given Rapa or Aca alone. If model II were correct, we might expect to see a lifespan effect, in females, that was not as strong as historical results for Rapa on its own, that is, reflecting negative effects of Aca not seen in male mice. The data also allow us to test the idea that the combination of two drugs produces a percentage or absolute lifespan extension greater than that produced by either agent alone in prior cohorts.

In male mice, the combination of Rapa and Aca produced a larger absolute and percentage change in survival than that seen in mice that had, in previous years, received the same dose (14 ppm) of Rapa alone. Table 5 collects statistics from the current study (RaAc9) for comparison to those of two prior Rapa‐only cohorts, set up in 2006 (Miller et al., 2011) and 2009 (Miller et al., 2014). Rapa‐treated males in the C2006 cohort lived, on average, 10% longer than simultaneous controls (average of values at each site), and C2009 males lived an average of 5% longer than controls. The RaAc9 males in the current study lived 19% longer (TJL and UM data; or 29% if UT mice are included), more than twice the percent change seen in either of the two earlier studies. Statistical comparison of the lifespan of the treated mice without reference to controls can eliminate concerns about abnormalities in the control lifespan, and the RaAc9 males lived an average of 949 days (average of three site‐specific medians), which is longer than the previous values of 887 and 852 days. Lastly, a site‐stratified log‐rank test, comparing RaAc9 mice to historical survival tables from C2006 to C2009, found a significant difference, with *p* < 0.006 and *p* < 0.0006 respectively. The log‐rank test does not involve any control data, and thus uses RaAc9 results from all three sites. The 90th percentile age for RaAc9 males is 21% higher than for controls (or 29% if one includes UT data), compared with changes of 16% in C2006 and 8% in C2009. Each of these comparisons leads to the conclusion that the combination of Rapa and Aca leads to a greater extension of male lifespan than the same dose of Rapa by itself, although caution is always warranted when comparing among data sets produced in different years.

**TABLE 5:** Comparison of RaAc9 data to historical Rapa‐only controls

	Males		Females	
Data Set	Median (Days) for RaAc9 or Rapa Mice	% Change in Median^a^	Median (Days) for RaAc9 or Rapa Mice	% Change in Median^a^
RaAc9 C2017	949 average TJL, UM, UT = 847, 987, 1014	19% (TJL + UM) 29% (3 sites)	1083 average TJL, UM, UT = 1147, 972, 1130	20% (TJL + UM) 27% (3 sites)
Rapa C2006	887 average TJL, UM, UT = 841, 932, 888	10%	1030 average TJL, UM, UT = 1077, 1008, 1006	18%
Rapa C2009	852 average TJL, UM, UT = 728, 974, 855	5%	1052 average TJL, UM, UT = 1002, 1065, 1089	18%

^a^ Mean of the two or three site‐specific medians when compared to simultaneous same‐sex controls.

The same set of comparisons for female mice suggests that the combination of Rapa plus Aca is neither distinctly better nor worse than would be expected in mice given Rapa only (see Table 5). In the C2006 and C2009 cohorts, Rapa increased female lifespan by 18% in each case. This is close to the 20% increase seen in RaAc mice from TJL plus UM (including the UT data implies a 27% increase). Comparison of the median levels (i.e., without regard to control mice) leads to a similar conclusion: the average median survival for RaAc9 was 1083, compared with 1030 for C2006 and 1052 for C2009 females. Lastly, a site‐stratified log‐rank test finds no significant difference in survival between RaAc9 females (all three sites) and C2006 females (*p* = 0.07) or C2009 females (*p* = 0.39). Thus, adding Aca to Rapa seems not to improve survival above that typically seen in Rapa‐treated mice, but neither does it diminish survival. Had we found that adding Aca to Rapa impairs survival with respect to mice given Rapa only, we could have concluded that Aca has negative effects that are especially strong in females, but the results indicate neither a harmful effect nor an additive effect beyond the expected effects of Rapa on its own. It is possible that the inability of Aca to improve survival in Rapa‐treated female mice could reflect either a weaker beneficial effect of Aca in females or a negative side effect, in females only, that opposes Aca benefits.

What might be the biological basis for the additive effect of combining acarbose and rapamycin in males and the absence of an additive effect in females? One rationale for combining the two drugs was the known insulin‐sensitizing effect of acarbose as an antidote to the insulin‐desensitizing effect of rapamycin, especially in males (Miller et al., 2014). Rapamycin is believed to extend lifespan by suppressing mTOR signaling, which has been implicated in aging across multiple species (Harrison et al., 2009). By contrast, acarbose, a glucosidase inhibitor known for reducing postprandial glucose surges and other anti‐diabetogenic effects, is likely to affect lifespan through pathways at least partly distinct from mTOR signaling, and these complementary mechanisms could account for the additive effects of the treatment. One such mechanism could be the salutary effects of acarbose on the microbiome. Analysis of the fecal microbiome and metabolites of acarbose‐treated mice from an earlier ITP cohort revealed shifts in microbiota and increases in short chain fatty acids that correlated with the increased lifespan of acarbose‐treated animals (Smith et al., 2019). Previously, metformin, although without efficacy when administered alone in the ITP trial, appeared to augment rapamycin's effect on longevity when the two drugs were combined (Strong et al., 2016), possibly by correcting the desensitizing effect of rapamycin on glucose regulation (Strong et al., 2016). The rapamycin/acarbose combination is thus the second treatment combination identified by the ITP that may be additive. Another group reported that combining a statin with an angiotensin receptor antagonist extended lifespan in male C3B6F1 hybrid mice, but either drug alone had no effect (Spindler et al., 2016). Together these results encourage further study of drug combinations, based on evidence for complementary or synergistic actions.

Interpretation of the RaAc16 data is on a less secure footing because there are no prior ITP studies of Rapa used alone for 16 months, and because of dramatic site‐specific variation in our current C2017 data. For females, it is clear that RaAc16 has a substantial lifespan benefit, producing a 12% increase in the median for the three‐site data and a 10% benefit when UT mice are omitted. Because we had previous shown (Miller et al., 2011) that Rapa started at 9 months had the same lifespan benefit as Rapa started at 20 months (in either sex), we anticipated that RaAc9 and RaAc16 would also produce similar results, but we found instead that the effects of RaAc16 on females were significantly less than those of RaAc9 (*p* = 0.0003 by site‐stratified log‐rank test; data from all three sites, since no control mice are needed for this comparison).

The effects of RaAc16 treatment on male mice varied greatly among sites, with a percent increase in a median of −1%, 2%, and 33% at TJL, UM, and UT respectively. Accordingly, the pooled effect was highly significant (14% increase, *p* < 0.001) using the standard three‐site analysis, but the more conservative two‐site analysis showed a much smaller survival effect on males (0% change, *p* = 0.03), with effects of RaAc16 restricted to mice over the age of 1000 days. A comparison of RaAc16 to RaAc9, using data from the RaAc‐treated mice only at all three sites, found that delaying the onset of drug treatment led to a much smaller survival benefit (*p* = 0.0002). We concluded that the combination of Rapa and Aca started at 16 months and led to a clear survival advantage in females and a less consistent effect in males. Since blood levels of Rapa are lower in males compared with females when the mice receive the same dose in chow, it would be informative to re‐test the effects of delayed drug onset when chow doses were adjusted to provide the same serum levels in mice of both sexes. We would predict that Rapa effects would be similar in both sexes if blood levels were equivalent, but do not have data to test this idea.

Captopril led to small (4% or 5%) but significant increases in female lifespan regardless of whether UT mice were included. In male mice, conclusions depend on the degree to which UT controls are considered a useful standard: *p* = 0.001 (14% increase) for the three‐site dataset, and *p* = 0.26 (6% increase) for the two‐site data. For male mice, the three sites had percent increases in a median of 11, −6, and 28% for TJL, UM, and UT, respectively. We view this as suggestive evidence for lifespan increases in captopril‐treated mice.

Captopril is the second ACE inhibitor to be tested by the ITP. The first, enalapril, did not affect survival (Harrison et al., 2009), but other ACE inhibitors have been reported to extend lifespan in male CF‐1 mice (Ferder et al., 1993) and female Wistar rats (Santos et al., 2009). Captopril was also found in a drug screen to increase lifespan in *C eleg*ans (Kumar et al., 2016). Given the evidence for its efficacy in this study, its clinical uses for hypertension, renal and liver disease, and the fact that other ACE inhibitors have extended rodent lifespan, follow‐up studies are warranted to establish replicability and if, replicable, optimal dosage.

BD led to a marginally significant effect in females when all three sites were included (*p* = 0.04 for a 1% change in median), which was not seen in the two‐site dataset (*p* = 0.3, 0% change in median). There was no significant effect on male survival and no significant effect in either sex for the longest surviving mice as evaluated by the Wang/Allison test. BD might produce clear effects at a different dose, but the current data provide almost no support for a lifespan benefit in UMHET3 mice.

Similarly, we found no evidence for lifespan benefits for leucine, PB125, Sulindac, or Syringaresinol, in either sex, at the doses and dose schedules used (See Tables S2 and S3).

The compounds that increased survival in this study, rapamycin‐acarbose, and captopril, were the only ones that significantly reduced body weight. Although we cannot exclude a role for weight reduction in lengthened lifespans, correlation does not imply causation. Moreover, in an earlier study, late‐life treatment with 14.7‐ppm rapamycin increased lifespan without reducing body weight (Harrison et al., 2009). Both NDGA and aspirin increased lifespan in male mice without reducing body weight (Strong et al., 2008). Similarly, in an earlier study, acarbose reduced weight more in females than in males, but increased survival more in males (Harrison et al., 2014). Thus, the longer survival of ACA‐treated males vs ACA‐treated females cannot by explained by proportionate sex differences in body weight. Finally, drugs that both increase survival and reduce body weight should not be discounted, given the dearth of interventions that achieve either.

The unexpected disparity in control mouse survival curves seen in C2017 presented unusual challenges for analysis and interpretation. We have tried to present our results in sufficient detail to allow readers to form their own opinions as to the strengths and weaknesses of the key conclusions, and we note that the raw survival datasets will be made available by the Mouse Phenome Database (https://phenome.jax.org/search?searchterm=ITP) to allow independent analytical approaches we did not consider. From our perspective, the difficulties encountered in the C2017 data show one of the benefits of testing each drug at three sites, so that differences among the sites can help to distinguish strong, consistent findings from those that are provocative but not entirely consistent. Conducting these studies over an extended period (from 2004 to the present) also provides us, and the scientific community more generally, with a rich set of historical results, on survival, weight, and pathology, against which to compare each new dataset as these come to fruition.

## 4 EXPERIMENTAL PROCEDURES

### 4.1 Animals

UM‐HET3 mice were produced and housed at each of the three test sites as previously described in detail (Strong et al., 2016; Harrison et al., 2014; Miller et al., 2011). Males were housed 3 per cage, while females were housed 4 per cage in individually ventilated cages (IVC).

At the age of 42 days, each cage was assigned to a control or test group by use of a random number table. Each mouse was then briefly anesthetized by isoflurane inhalation administered either by nose cone or by an instrument designed for small animal anesthesia and a radio‐frequency identification chip was implanted by sterile syringe. The duration of anesthesia was approximately 1–2 min.

Mice received Purina 5LG6 control diet until treatments were begun. Starting at various ages as listed in the results section, mice in the treatment groups received Purina 5LG6 containing the additives, at all three sites, and mice in the control group received Purina 5LG6 without additives.

Details of the methods used for health monitoring were provided in Miller et al., (2014); in brief, each of the three colonies was evaluated four times each year for infectious agents, including pinworm. All such tests were negative throughout the entire study period.

### 4.2 Control and experimental diets

All diets were prepared by Test Diet, Inc., a division of Purina Mills. Purina 5LG6 mouse chow containing each of the test substances, and batches of the control diet, were prepared at intervals of approximately 4 months. Each batch of food was shipped at the same time to each of the three test sites. Samples of diet from each batch were tested for recovery (% of compound ppm added to the diet). Detailed protocols for measuring compounds are provided in Appendix I (Supplementary Information). Table S4 lists the average recovery of each compound, averaged across the batches tested. Young adult female and male test mice were administered each diet. After 8 weeks, blood was collected and plasma concentrations of each compound were measured. The results of these measurements are shown in Table S5. (R/S)‐1,3‐butanediol was obtained from Sigma Aldrich Chemical Company. It was mixed with chow at a concentration of 100,000 ppm (milligrams per kilogram of food) and fed to mice beginning at 6 months of age. Plasma levels are reported for BD itself, not for putative active metabolites such as R‐β‐hydroxybutyrate. Syringaresinol was a gift from Dr. Yousin Suh, Columbia University, and obtained from AMOREPACIFIC. It was mixed with chow at a concentration of 300 ppm and fed to mice beginning at 5 months of age. Sulindac was obtained from the pharmacy at the University of Michigan. It was mixed with chow at a concentration of 5 ppm and fed to mice beginning at 5 months of age. Captopril was purchased from Sigma Aldrich. It was mixed with chow at a concentration of 180 ppm and fed to mice beginning at 5 months of age. L‐Leucine was obtained from Chem Impex International. It was combined with mouse chow at a concentration of 40,000 ppm and fed to mice starting at 5 months of age. PB125 was obtained from Pathways Bioscience who formulate it to contain specified amounts of the phytochemicals: carnosol, withaferin A and luteolin. It was mixed with mouse chow at 4.7 ppm carnosol, 9 ppm luteolin, and 0.47 ppm withaferin A. Treatment with the PB125‐laced diet started at 5 months of age. Encapsulated rapamycin was obtained from Emtora Biosciences (formerly Rapamycin Holdings). Acarbose, purchased from Spectrum Chemical Mfg. Corp., Rapamycin, and acarbose were mixed in the diet at a concentration of 14.7 ppm and 1000 ppm, respectively, and treatment was started in one group at 9 months and another group at 16 months of age and continued until death.

### 4.3 Removal of mice from the longevity population

As described in detail in Miller et al., 2011, mice were removed from the study because of illness, fighting, or accidental death, typically during chip implantation, because of chip failure, or water bottle leakage, or because they were used for another experimental purpose, such as testing for blood levels of a test agent (see Table S4). For survival analyses, all such mice were treated as alive at the date of their removal from the protocol and lost to follow‐up thereafter. These mice were not included in calculations of median longevity.

### 4.4 Estimation of age at death (lifespan)

Mice were examined twice daily for signs of ill health. Mice were euthanized for humane reasons if so severely moribund that they were considered, by an experienced technician, unlikely to survive for more than an additional 48 hrs. A mouse was considered severely moribund if it exhibited more than one of the following clinical signs: (a) inability to eat or to drink; (b) severe lethargy, as indicated by a reluctance to move when gently prodded with a forceps; (c) severe balance or gait disturbance; (d) rapid weight loss over one week or more; or (e) an ulcerated or bleeding tumor. The age at which a moribund mouse was euthanized was taken as the best available estimate of its natural lifespan. Mice found dead were also noted at each daily inspection.

### 4.5 Statistical methods

The statistical analysis plan was specified before initiating the study and has been followed for all ITP studies. For each sex, we performed site‐specific and combined site analyses. We calculated the median survival for the control group as well as for each treatment group. To compute the median percentage increase, we subtracted the median age in the control group from the corresponding value in the treatment group and divided the difference by the median age of the control group and multiplied by 100. Using a two‐sided 5% significance level, we performed the log‐rank test to determine whether survival curves for mice receiving treatment differ from the survival function for control mice. Log‐rank tests that pooled data across the three test sites used a method that stratifies by the site. To assess the maximum life span, we computed the 90th percentile age of both the treated and control mice. To determine which treatments prolonged longevity in mice, we utilized the Wang‐Allison test (Wang et al., 2004). This is the Fisher Exact Test comparing the numbers of mice surviving in the control and treatment group at the age corresponding to the 90th percentile of lifespan in the joint survival distribution. We further assessed the longevity of mice in all three sites combined using a modified version of the Wang–Allison test in the manner with which the 2 × 2 contingency table is constructed separately for each site. We report the sum of corresponding site‐specific 2 × 2 table cell entries as the combined site 2 × 2 table cell entries. This allows for information from all sites to be used in a balanced manner.

## AUTHOR CONTRIBUTIONS

R.S., R.A.M., and D.E.H. are the principal investigators at the three collaborating institutions and are responsible for project design, supervision of technical personnel, interpretation of results, and preparation of manuscript drafts. N.A.R., D.B.L., J.F.N., and A.B.S. participated in the preparation of manuscript drafts and provided advice on experimental design and interpretation. E.F. and V.D. supervised animal laboratory personnel and data collection at UT. P.R. supervised animal laboratory personnel and data collection at T.J.L., M.A.J, B.G., and M.L.C. supervised analytical pharmacology studies at U.T., M.A.B., C.J.C., J.G., A.G., and S.K. assisted with data analysis. M.K. and P.R. proposed the rapamycin plus acarbose intervention. K.K. proposed the captopril intervention. J.A.D. and A.O. proposed the leucine intervention. K.L H. and B.F.M. proposed the PB125 intervention. H.W. and S.K.A. proposed the Sulindac intervention. Y.S. proposed the Syringaresinol intervention. J.N. and E.V. proposed the 1,3‐butanediol intervention. M.F. served as the project officer for the National Institute on Aging and contributed to program development, experimental design, and analysis.

## CONFLICT OF INTEREST

The University of Texas Health Science Center at San Antonio has a patent, by inventor Randy Strong, for an encapsulated rapamycin formulation used in the research described in this paper. Under a licensing agreement between Emtora Biosciences and the University of Texas Health Science Center San Antonio, R. Strong and Z.D. Sharp (co‐Inventor), the University is entitled to milestone payments and royalty on sales of microencapsulated rapamycin. The university has a plan for managing conflicts of interest under its “Policy and Procedures for Promoting Objectivity in Research by Managing, Reducing or Eliminating Conflicts of Interest”.

## Supporting information


Data S1

## Data Availability

The data that support the findings of this study are openly available in The Jackson Laboratory Mouse Phenome Database at https://phenome.jax.org/search?searchterm=ITP.
