# Supplementary material for: Lifespan benefits for the combination of rapamycin plus acarbose and for captopril in genetically heterogeneous mice
Source: Aging Cell. 2022 Sep 30;21(12):e13724. doi: 10.1111/acel.13724 (PMC9741502; doi:10.1111/acel.13724)
Supplement: Supplementary file 1 — Data S1 [file ACEL-21-e13724-s001.docx]

**Supplemental Figures S1 – S5**

**Supplemental Figure 1**


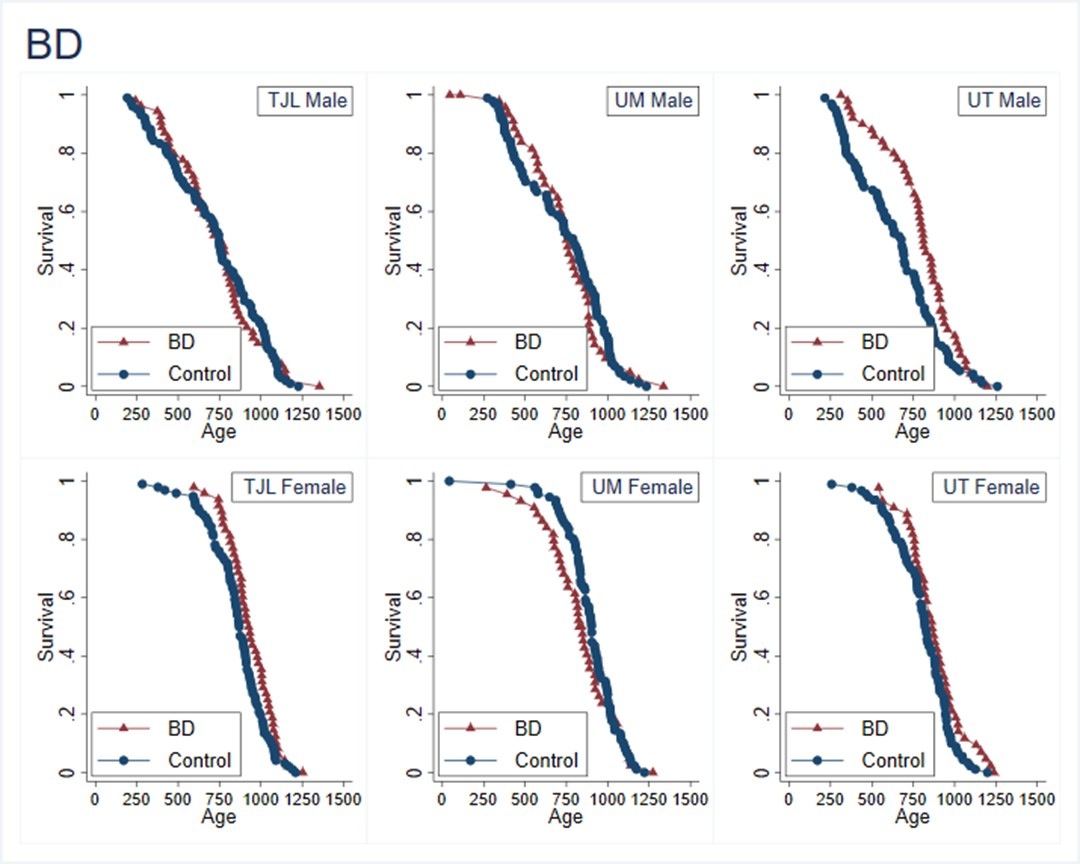


**Supplemental Figure 2**


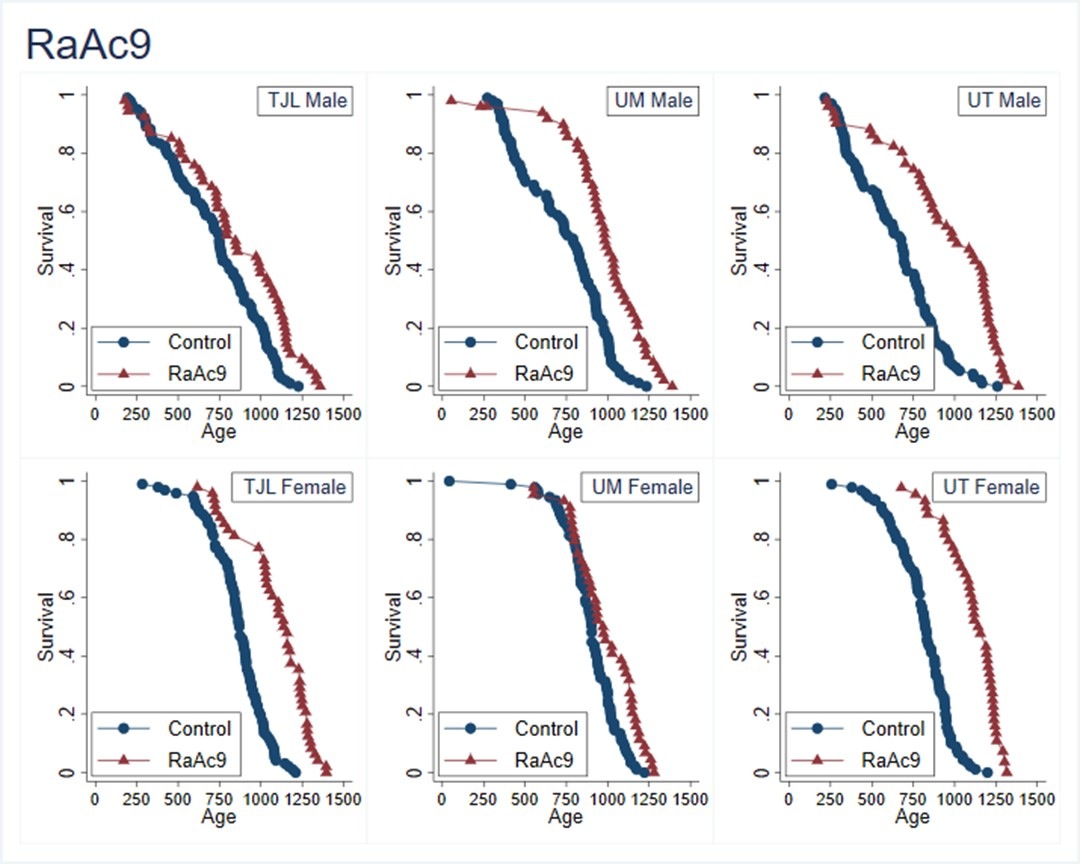


**Supplemental Figure 3**


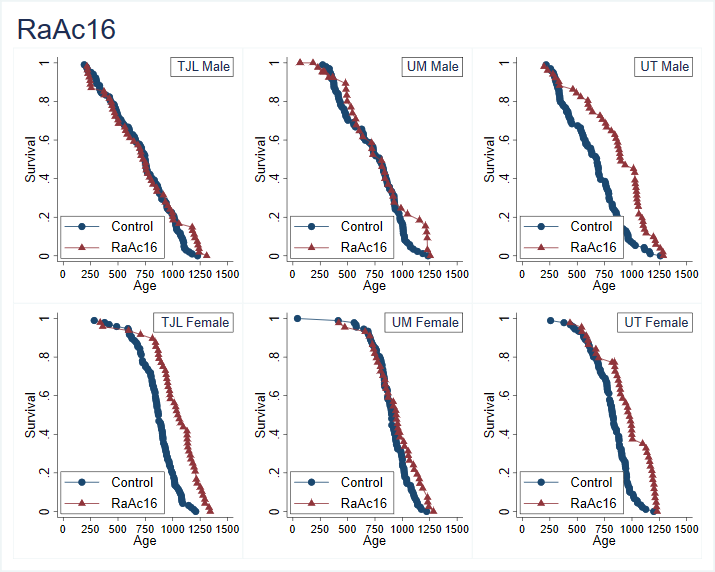


**Supplemental Figure 4**


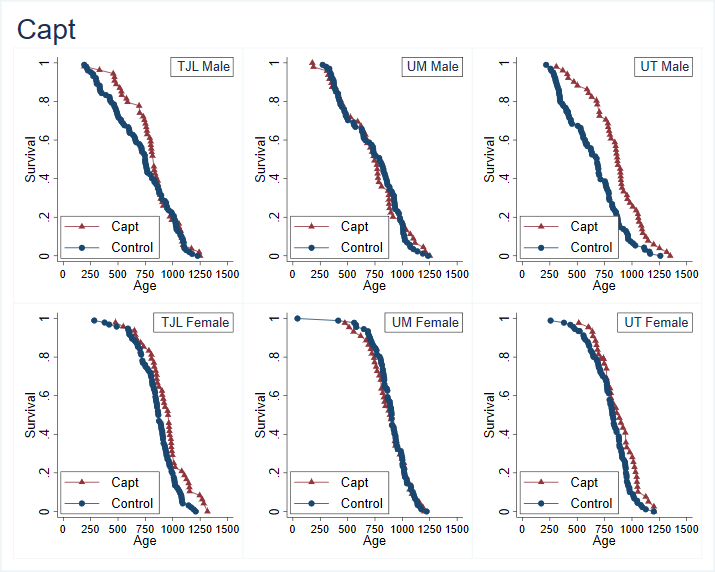


**Supplemental Figure 5**


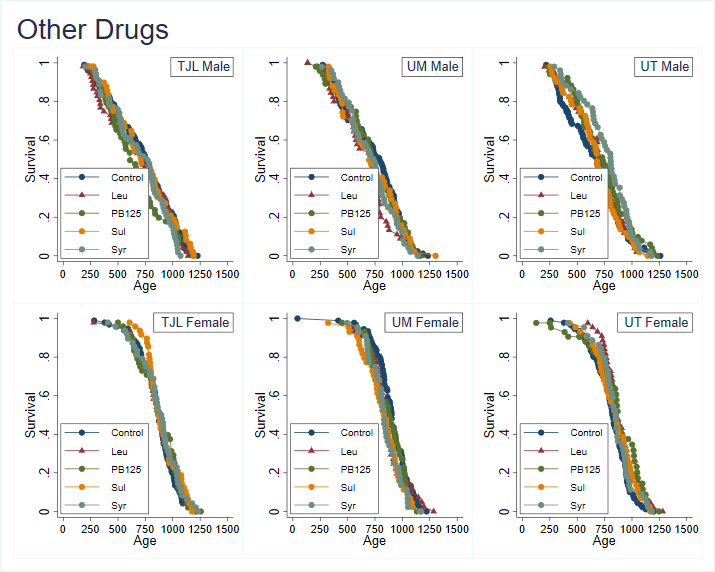


**Supplemental Tables:**

**Table S1: Summary of the median lifespan of ITP control mice, by site, C2004 – C2017**

|  |  |  |  |  |  |  |
| --- | --- | --- | --- | --- | --- | --- |
| **Cohort** | **TJL male** | **UM male** | **UT male** | **TJL female** | **UM female** | **UT female** |
| C2004 | 757 | 865 | 739 | 858 | 916 | 880 |
| C2005 | 803 | 895 | 722 | 881 | 878 | 896 |
| C2006 | 800 | 851 | 813 | 889 | 891 | 843 |
| C2007 | 728 | 793 | 810 | 860 | 900 | 840 |
| C2009 | 807 | 925 | 704 | 894 | 887 | 866 |
| C2010 | 683 | 926 | 732 | 864 | 931 | 906 |
| C2011 | 763 | 861 | 715 | 892 | 872 | 857 |
| C2012 | 844 | 872 | 751 | 924 | 843 | 873 |
| C2013 | 803 | 912 | 807 | 890 | 870 | 897 |
| C2014 | 873 | 894 | 743 | 904 | 884 | 882 |
| C2015 | 819 | 740 | 786 | 928 | 899 | 892 |
| C2016 | 752 | 826 | 799 | 883 | 858 | 882 |
|  |  |  |  |  |  |  |
| Mean | 786 | 863 | 760 | 889 | 886 | 876 |
| SD | 52 | 55 | 40 | 22 | 24 | 21 |
| CoV | 7% | 6% | 5% | 3% | 3% | 2% |
| SEM | 15 | 16 | 12 | 6 | 7 | 6 |
|  |  |  |  |  |  |  |
| C2017 | 748 | 803 | 676 | 873 | 902 | 820 |
| Z-score | -0.73 | -1.09 | -2.1 | -0.73 | 0.67 | -2.67 |
| p-value for Z-score | 0.2 | 0.1 | 0.018 | 0.2 | 0.7 | 0.0038 |

The Table shows the median lifespan of control mice at each ITP site, for each sex, in cohorts born in each year from 2004 to 2016. The bottom rows show the mean value for each site/sex, standard deviation (SD), coefficient of variation (CoV = SD/mean as a percentage), and standard error of the mean (SEM). Corresponding data for C2017 are included but were not used to calculate the tabulated statistics, along with the Z-score (standard deviations above or below the mean for C2004—C2016), and one-tailed p-values for the C2017 control lifespans. Supplemental Methods

**Table S2. Recovery of compounds added to the diet averaged over the batches sent during the study**. Methods for measuring each compound are described below.

|  |  |  | |  |  |
| --- | --- | --- | --- | --- | --- |
|  |  |  |  | Food (PPM) |  |
| Intervention | Constituent | Mean | S.D. | Expected | % Expected |
|  |  |  |  |  |  |
| (R/S)-1,3-Butanediol |  | 88300 | 2060 | 100,000 | 88% |
|  |  |  |  |  |  |
| Captopril |  | 39.8 | 23.8 | 180 | 22% |
|  |  |  |  |  |  |
| Leucine | Treated | 40900 | 4100 | 40,000 | 102% |
|  | Control | N/A |  |  |  |
|  |  |  |  |  |  |
| PB125* | Carnisol | 3.2 | 2.8 | 4.7 | 67% |
|  | Luteolin | 4.8 | 4.6 | 9 | 54% |
|  | Withaferin A | 1.0 | 3.2 | 0.47 | 206% |
|  |  |  |  |  |  |
| Rapamycin + Acarbose | Rapamycin | 12.3 | 4.2 | 14.7 | 84% |
|  | Acarbose | 159.0 | 83.6 | 1000 | 16% |
|  |  |  |  |  |  |
| Sulindac | Sulindac | 3.2 | 0.9 | 5 | 63% |
|  | Sulindac Sulfone | N/A |  |  |  |
|  | Sulindac Sulfide | N/A |  |  |  |
|  |  |  |  |  |  |
| **Syringaresinol*** |  | 323 | 38 | 300 | 108% |

**Table S3 Plasma concentrations of compounds.** Prior to initiation of each study, young adult test mice were given the diet and after 8 weeks on the diet, plasma was collected, and compounds measured. Methods for measuring each compound are described below.

|  | | **Female Plasma** | | | | | **Male Plasma** | | | | |  |
| --- | --- | --- | --- | --- | --- | --- | --- | --- | --- | --- | --- | --- |
| **Intervention** | **Treatment** | **Min** | ***M*** | ***Mdn.*** | **Max** | **N** | **Min** | ***M*** | ***Mdn.*** | **Max** | **N** | **P value** |
| **But [µg/ml)]** | **Treated** | 1.78 | 2.64 | 2.49 | 3.81 | 4 | 9.09 | 19.28 | 23.6 | 25.2 | 3 | 0.057 |
|  | **Control** | <LOD | <LOD | <LOD | <LOD | 5 | <LOD | <LOD | <LOD | <LOD | 2 | N/A |
| **Capto [ng/ml]** | **Treated** | 142 | 234 | 213 | 368 | 4 | 106 | 126 | 121 | 151 | 3 | 0.114 |
|  | **Control** | <LOD | <LOD | <LOD | <LOD | 4 | <LOD | <LOD | <LOD | <LOD | 3 | N/A |
| **Leucine [µg/ml]** | **Treated** | 61.0 | 68.4 | 68.0 | 76.6 | 4 | 109 | 136 | 137 | 161 | 3 | 0.0002 |
|  | **Control** | 53.4 | 56.6 | 56.4 | 60.4 | 4 | 57.2 | 60.6 | 60.3 | 64.4 | 3 | 0.976 |
| **Carn [ng/ml] (PB125)** | **Treated** | *0 | *13.0 | 15.6 | 22.1 | 8 | 22.3 | *29.1 | *30.5 | 35.2 | ^α^6 | 0.0047 |
|  | **Control** | *0 | *2.59 | *0 | 18.1 | 7 | *0 | *7.37 | *5.30 | 19.5 | ^α^6 | 0.695 |
| **Lut [ng/ml] (PB125)** | **Treated** | <LOD | <LOD | <LOD | <LOD | 8 | <LOD | <LOD | <LOD | <LOD | ^α^6 | N/A |
|  | **Control** | <LOD | <LOD | <LOD | <LOD | 7 | <LOD | <LOD | <LOD | <LOD | ^α^6 | N/A |
| **WA [ng/ml] (PB125)** | **Treated** | <LOD | <LOD | <LOD | <LOD | 8 | <LOD | <LOD | <LOD | <LOD | ^α^6 | N/A |
|  | **Control** | <LOD | <LOD | <LOD | <LOD | 7 | <LOD | <LOD | <LOD | <LOD | ^α^6 | N/A |
| **Acarb [ng/ml] (Rapa + Acar)** | **Treated** | 17.9 | 29.9 | 27.6 | 46.5 | 4 | 4.57 | 9.66 | 10.6 | 13.8 | 3 | 0.057 |
|  | **Control** | <LOD | <LOD | <LOD | <LOD | 4 | <LOD | <LOD | <LOD | <LOD | 3 | N/A |
| **Rapa [ng/ml] (Rapa + Acar)** | **Treated** | 70.9 | 92.1 | 73.7 | 150 | 4 | 39.5 | 108 | 41.9 | 242 | 3 | 0.629 |
|  | **Control** | <LOD | <LOD | <LOD | LOD | 4 | <LOD | <LOD | <LOD | <LOD | 3 | N/A |
| **Sulindac [µg/ml]** | **Treated** | 0.221 | 0.261 | 0.258 | 0.308 | ^ß^4 | 0.158 | 0.393 | 0.233 | 0.787 | ^ß^3 | 0.908 |
|  | **Control** | *0 | *0.171 | *0.210 | 0.265 | ^ß^4 | 0.198 | 0.229 | 0.210 | 0.280 | ^ß^3 | 0.999 |
| **S-Sulfone [µg/ml]** | **Treated** | 0.091 | 0.140 | 0.151 | 0.168 | ^ß^4 | 0.195 | 0.423 | 0.528 | 0.547 | ^ß^3 | 0.187 |
|  | **Control** | *0 | *0.445 | *0.0190 | 0.140 | ^ß^4 | 0.158 | 0.181 | 0.189 | 0.196 | ^ß^3 | 0.892 |
| **S-Sulfide [µg/ml]** | **Treated** | 0.0870 | 0.0985 | 0.0975 | 0.108 | ^ß^4 | *0 | *0.0710 | *0.102 | 0.111 | ^ß^3 | 0.840 |
|  | **Control** | <LOD | <LOD | <LOD | <LOD | ^ß^4 | <LOD | <LOD | <LOD | <LOD | ^ß^3 | 0.839 |
| **Syringaresinol [ng/ml]** | **Treated** | *0 | 7.59 | 7.68 | 16.2 | 8 | *0 | 39.2 | 7.90 | 108 | 6 | 0.659 |
|  | **Control** | <LOD | <LOD | <LOD | <LOD | 7 | <LOD | <LOD | <LOD | <LOD | 7 | N/A |

Abbreviations: But = (R/S)-1,3-butanediol; Capto = captopril; Carn = carnosol; Lut = luteolin; WA = withaferin A; acarb = Acarbose; Rapa = rapamycin; S-Sulfone = sulindac sulfone; S-Sulfine = sulindac sulfide. LOD = Limit of Detection. *M* = mean; *Mdn* = median.

* These numbers are values below the LOD written as 0 for statistical purposes. The means and medians were calculated using zeros for those values below the LOD.

^α^Carnosol, luteolin, and withaferin A, in PB125, concentrations were analyzed in the plasma of 8 treated females, 6 treated male, 7 control female, and 6 control male mice.

^ß^Sulidac and its metabolites, sulindac sulfone, and sulindac sulfide concentrations were analyzed in 3 treated female, 3 treated male, 3 control female, and 3 control male mice.

Interventions in which control sample levels were below the LOD were statistically analyzed using a non-parametric Mann-Whitney test ((R/S)-1,3-butanediol, captopril, rapamycin, acarbose, and syringaresinol). Leucine and carnosol concentrations were analyzed using an ordinary two-way ANOVA. Sulindac, sulindac sulfone, and sulindac sulfide concentrations were analyzed using a three-way ANOVA. All statistical analyses were performed using GraphPad Prism, version 9.4.0 (GraphPad Software, San Diego, CA).

(R/S)-1,3-butanediol LOD ≤ 0.005 µg/ml; captopril LOD ≤ 10 ng/ml; carnosol LOD ≤ 5.0 ng/ml; luteolin LOD ≤ 5 ng/ml; withaferin A LOD ≤ 5 ng/ml; acarbose LOD < 3.0 ng/ml; rapamycin LOD < 1.5 ng/ml; sulindac LOD ≤ 0.001 µg/ml; sulindac sulfone LOD ≤ 0.001 µg/ml; sulindac sulfide LOD ≤ 0.001 µg/ml; syringaresinol LOD ≤ 5.0 ng/ml.

|  |  |  |  |  |  |  |
| --- | --- | --- | --- | --- | --- | --- |

**Table S4.** The number of male or female mice euthanized for humane reasons compared to the total number at each site and intervention.

| **Group** |  | **Fight_UT** | **Accident_UT** | **Illness_UT** | **Fight_UM** | **Accident_UM** | **Illness_UM** | **Fight_TJL** | **Accident_TJL** | **Illness_TJL** |
| --- | --- | --- | --- | --- | --- | --- | --- | --- | --- | --- |
| **Control** M |  | 2 | 2 | 0 | 8 | 0 | 0 | 0 | 0 | 0 |
| **Control** F |  | 0 | 2 | 1 | 0 | 1 | 1 | 0 | 0 | 0 |
| **PB125** M |  | 0 | 0 | 1 | 9 | 0 | 0 | 3 | 0 | 0 |
| **PB125** F |  | 0 | 8 | 0 | 0 | 0 | 0 | 0 | 0 | 0 |
| **Sul** M |  | 0 | 0 | 2 | 6 | 0 | 0 | 6 | 0 | 0 |
| **Sul** F |  | 0 | 0 | 1 | 0 | 0 | 0 | 0 | 0 | 0 |
| **BD** M |  | 0 | 2 | 1 | 8 | 1 | 0 | 0 | 0 | 0 |
| **BD** F |  | 0 | 1 | 4 | 0 | 0 | 1 | 0 | 0 | 0 |
| **Leu** M |  | 0 | 0 | 1 | 6 | 0 | 0 | 3 | 0 | 0 |
| **Leu** F |  | 0 | 0 | 2 | 0 | 0 | 0 | 0 | 0 | 0 |
| **Capt** M |  | 0 | 0 | 0 | 3 | 0 | 0 | 0 | 0 | 0 |
| **Capt** F |  | 0 | 4 | 0 | 0 | 0 | 0 | 0 | 0 | 0 |
| **Syr** M |  | 0 | 0 | 0 | 3 | 0 | 0 | 0 | 1 | 0 |
| **Syr** F |  | 0 | 0 | 1 | 0 | 0 | 0 | 0 | 0 | 0 |
| **RaAc9** M |  | 0 | 0 | 0 | 3 | 0 | 0 | 0 | 0 | 0 |
| **RaAc9** F |  | 0 | 2 | 0 | 0 | 0 | 0 | 0 | 0 | 0 |
| **RaAc16** M |  | 0 | 0 | 1 | 12 | 0 | 0 | 0 | 0 | 0 |
| **RaAc16** F |  | 0 | 1 | 1 | 0 | 0 | 1 | 0 | 0 | 0 |

The number of mice of either sex that were euthanatized at each site for fighting; illness; or accident (e.g. water bottle leakage).

Appendix I. Supplemental Methods

Measurement of Butanediol in Mouse Food using HPLC/MS/MS

(R/S)-1,3- Butanediol (BD) was purchased from Sigma Aldrich (St. Louis, MO). All other reagents were purchased from Thermo Fisher Scientific (Waltham, MA). Milli-Q water was used for the preparation of all solutions. The HPLC/MS/MS system consisted of a Shimadzu SIL 20A HT autosampler, LC-20AD pumps (2), and an AB Sciex API 3200 tandem mass spectrometer with turbo ion spray. The LC analytical column was an ACE Excel C18 (75 x 3.0 mm, 5 microns) purchased from Mac-Mod Analytical (Chaddsford, PA). Mobile phase A contained 20mM ammonium formate dissolved in water at a pH of 3.5. Mobile phase B contained 100% HPLC grade acetonitrile. The initial mobile phase was 20% B and at 1 minute after injection was ramped to 95% B. From 3.0 min to 5.0 min the mobile phase was maintained at 95% B and 5.1 minutes was switched immediately back to 20% B and ran for 4.9 minutes to equilibrate the column before the next injection. The transition used was 91.04 🡪 55.1 in positive mode. 1,3-butanediol eluted at 1.6 min.

Pure BD from Sigma was used to spike the food calibrator samples. Food calibrator samples were prepared at concentrations of 0, 1.25, 2.5, 5, 10, and 20% BD. BD was quantified in mouse food by mixing 100 mg of the calibrator and unknown samples with 1 mL of 90/10 mobile phase B/mobile phase A and then shaking for 30 minutes. The samples were then centrifuged for 10 minutes at 3,200 g. 10 µl of the supernatant was added to 490 µl of 90/10 MP B/MP A. This was vortexed and then transferred to autosampler vials. 2 µL was injected into the HPLC/MS/MS. The peak area ratios for each unknown sample were compared against a linear regression of calibrator peak area ratios to quantify BD. The concentration of BD was expressed as % of the food.

Measurement of Captopril in Mouse Food Using HPLC-UV

Captopril was purchased from Sigma-Aldrich Chemical Company (St. Louis, MO). All other reagents were purchased from Thermo Fisher Scientific (Waltham, MA). Milli-Q water was used for the preparation of all solutions. The HPLC/UV system consisted of a Shimadzu SIL 20A HT autosampler, LC-20AD pumps (2), and an SPD-20A UV detector. The LC analytical column was an ACE Excel C18-AR (75 x 3.0 mm, 3 microns) purchased from Mac-Mod Analytical (Chaddsford, PA) and was maintained at 45^o^C during the chromatographic runs using a Shimadzu CT-20A column oven. The mobile phase contained 30% HPLC grade methanol, pH 3.0. The flow rate of the mobile phase was 0.5 ml/min. Captopril eluted at 3.25 minutes.

Captopril stock solution was prepared in methanol at a concentration of 1 mg/ml and stored in aliquots at -80^o^C. A working stock solution was prepared each day from the super stock solutions at a concentration of 100 μg/ml and used to spike the calibrators. Food calibrator samples were prepared by spiking food samples at concentrations of 0, 25, 50, 100, and 200 ng/mg. Captopril was quantified in mouse food by mixing 100 mg of the calibrator and unknown samples with 1 mL of methanol and then shaking for 30 minutes. The samples were then centrifuged for 10 minutes at 3,200 g. The supernatant was transferred to microcentrifuge tubes and spun at 17,000 g for 2 minutes. The final extracts were transferred to autosampler vials and 10 µL was injected into the HPLC/UV. The peak area ratios for each unknown sample were compared against a linear regression of calibrator peak area ratios to quantify captopril. The concentration of captopril was expressed as ng/mg or ppm of food.

Measurement of Leucine Using HPLC-EC

Leucine, Valine, and all reagents were purchased from Sigma Chemical Company (St. Louis, MO). Milli-Q water was used for the preparation of all solutions. The HPLC system consisted of an ESA Model 542 autosampler, an ESA Model 584 pump, and a Coulochem III electrochemical detector with an ESA 5014B electrochemical cell and a 5020 Guard cell. The LC analytical column was an Alltima C18 Excel (150mm X 4.6mm 5 microns. The mobile phase contained 50% methanol, 49% Millipore water, 0.35% phosphoric acid, and 0.05% mM EDTA. The pH of the mobile phase was adjusted to 6.7 using 8N sodium hydroxide. The flow rate of the mobile phase was 0.8 ml/min. The detector settings were D1 at +150 mV, R1 at 10uA, and D2 at +550 mV, R2 at 10uA. Leucine eluted at 29 minutes.

Leucine super stock solutions were prepared in Milli-Q water at a concentration of 1 mg/ml and stored in aliquots at -80^o^C. Working stock solutions were prepared each day from the super stock solutions at concentrations of 100 and 10 μg/ml and used to spike the calibrators. An o-phthaldialdehyde derivatizing reagent (o-PA) was prepared by dissolving 27 mg pthaldialdehyde in 1 ml methanol, then adding 9 ml of a saturated sodium borate solution (pH 9) and finally adding 5 µL of β-mercaptoethanol. Each day 10 mL of a fresh derivatizing solution which consisted of 2.5 mL o-PA reagent and 7.5 mL saturated sodium borate (pH 9) was loaded into the autosampler.

Food calibrator samples were prepared by spiking food samples at concentrations of 0, 2, 4, 6, and 8%. Leucine was quantified in mouse food by mixing 10 mg of the calibrator and unknown samples with 30 µL of 1 mg/mL valine and 10 mL of Milli-Q water. The samples were then vortexed vigorously and shaken for 20 minutes. Then 100 µL of each sample was mixed with 0.9 ml Milli-Q water in microfilterfuge tubes, vortexed, and spun at 13,000 g for 1 minute. The final extracts were transferred to autosampler vials, and the injections were made by adding 15 µL of the derivatizing solution to the samples and injecting 27 µL into the HPLC system. The ratios of leucine peak areas to valine peak areas for each unknown sample were compared against a linear regression of the ratios obtained by the calibration samples to quantify leucine. The concentration of leucine was expressed as % of leucine in food.

Measurement of Rapamycin Using HPLC-tandem MS

Rapamycin, Ascomycin, and all reagents were purchased from Sigma Chemical Company (St. Louis, MO). Milli-Q water was used for the preparation of all solutions. The HPLC system consisted of a Shimadzu SIL 20A HT autosampler, LC-20AD pumps (2), and an AB Sciex API 3200 tandem mass spectrometer with turbo ion spray. The LC analytical column was a Grace Alltima C18 (4.6 x 150 mm, 5 microns) purchased from Alltech (Deerfield, IL) and was maintained at 25^o^C during the chromatographic runs using a Shimadzu CT-20A column oven. Mobile phase A contained 10 mM ammonium formate and 0.1% formic acid dissolved in 100% HPLC grade methanol. Mobile phase B contained 10 mM ammonium formate and 0.1% formic acid dissolved in 90% HPLC-grade methanol. The flow rate of the mobile phase was 0.5 ml/min. Rapamycin was eluted with a gradient. The initial mobile phase was 100% B and at 0.10 minutes after injection was ramped to 100% A. From 4.0 min to 5.0 min the mobile phase was maintained at 100% A and 5.1 minutes was switched immediately back to 100% B and ran for 4.9 minutes to equilibrate the column before the next injection. The Rapamycin transition was detected in positive mode at 931.6 Da 🡪 864.5 Da. The internal standard (Ascomycin) transition was 809.6 Da 🡪 756.6 Da. Rapamycin eluted at 5.6 min.

Rapamycin super stock solutions were prepared in methanol at a concentration of 1 mg/ml and stored in aliquots at -80^o^C. A working stock solution was prepared each day from the super stock solutions at a concentration of 10 μg/ml and used to spike the calibrators. Food calibrator samples were prepared by spiking food samples at concentrations of 0, 100, 500, 1000, and 2000 ng/mg. Rapamycin was quantified in mouse food by mixing 20 mg of the calibrator and unknown samples with 5 µL of 1 mg/mL Ascomycin and 4 mL of mobile phase A then vortexing vigorously and shaking for 20 minutes. Then 300 µL of the solution were transferred to microfilterfuge tubes and spun at 13,000 g for 1 minute. The final extracts were transferred to autosampler vials and 10 µL were injected into the LC/MS/MS. The peak area ratios for each unknown sample were compared against a linear regression of calibrator peak area ratios to quantify Rapamycin. The concentration of Rapamycin was expressed as ng/mg of food.

Measurement of Acarbose Using HPLC-tandem MS

Acarbose was obtained from Sigma Chemical Company (St. Louis, MO). HPLC grade methanol was purchased from Fisher (Fair Lawn, NJ). All other reagents were purchased from Sigma Chemical Company (St. Louis, MO). Milli-Q water was used for the preparation of all solutions. The HPLC system consisted of a Shimadzu SIL 20A HT autosampler, LC-20AD pumps (2), and an AB Sciex API 3200 tandem mass spectrometer with turbo ion spray. The LC analytical column was a Grace Alltima C18 (4.6 x 150 mm, 5 microns) purchased from Alltech (Deerfield, IL) and was maintained at 25^o^C during the chromatographic runs using a Shimadzu CT-20A column oven. Mobile phase A contained 10 mM ammonium formate and 0.1% formic acid dissolved in HPLC grade methanol. Mobile phase B contained 10 mM ammonium formate and 0.1% formic acid dissolved in 90% HPLC-grade methanol. The flow rate of the mobile phase was 0.4 ml/min. Acarbose was eluted with a gradient. The initial mobile phase was 100% B and at 0.10 minutes after injection was ramped to 100% A. From 4.0 min to 5.0 min the mobile phase was maintained at 100% A and 5.1 minutes was switched immediately back to 100% B and ran for 4.9 minutes to equilibrate the column before the next injection. The Acarbose transition was detected at 646.03 🡪 304.20 Da. Acarbose eluted at 4 min

Acarbose super stock solutions were prepared in methanol at a concentration of 1 mg/ml and stored in aliquots at -80^o^C. A working stock solution was prepared each day from the super stock solutions at a concentration of 10 μg/ml and used to spike the calibrators. Food calibrator samples were prepared by spiking food samples at concentrations of 0, 100, 500, 1000, and 2000 ng/mg. Acarbose was quantified in mouse food by mixing 20 mg of the calibrator and unknown samples with 4 mL of mobile phase A then vortexing vigorously and shaking for 10 minutes. Then 300 µL of the solution were transferred to microfilterfuge tubes and spun at 13,000 g for 1 min and then transferred to autosampler vials where 10 µL were injected into the LC/MS/MS. The peak area response of acarbose for each unknown sample was compared against a linear regression of calibrator response peak areas to quantify acarbose. The concentration of acarbose was expressed as ng/mg of food.

Measurement of PB125 in Mouse Food and serum using LC/MS/MS

The constituents of PB125 were luteolin (LUT), withaferin A (WFA), and carnosol (CRN). Reference standards of LUT and WFA were purchased from Sigma Aldrich (St. Louis, MO) while CRN was purchased from Cayman Chemical (Ann Arbor, MI). Cannabidiol (CBD), the internal standard for negative mode serum was also purchased from Sigma Aldrich. Mito Q, the internal standard for positive mode serum was obtained from the Jackson Lab (Bar Harbor, ME). All other reagents were purchased from Thermo Fisher Scientific (Waltham, MA). HPLC grade methanol was used for the preparation of all solutions.

The HPLC/MS/MS system consisted of a Shimadzu SIL 20A HT autosampler, LC-20AD pumps (2), and an AB Sciex API 3200 tandem mass spectrometer with turbo ion spray. The LC analytical column was an ACE Excel C18 (75 x 3.0 mm, 3 microns) purchased from Mac-Mod Analytical (Chaddsford, PA). Mobile phase A contained 0.1% formic acid dissolved in water. Mobile phase B contained 0.1% formic acid dissolved in 100% HPLC grade acetonitrile. The initial mobile phase was 25% B and 2 minutes after injection was ramped to 99% B. From 6.0 min to 10.0 min the mobile phase was maintained at 99% B and 10.01 minutes was switched immediately back to 25% B and ran for 2.09 minutes to equilibrate the column before the next injection. The LUT and CRN transitions were detected in negative mode, respectively at 285 🡪 132.9 Da and 329 🡪 285 Da. The WFA transition was detected in positive mode at 471 🡪 281 Da. LUT eluted at 5.2 min, CRN eluted at 7.5 min and WFA eluted at 6.2 min.

LUT, CRN, and WFA stock solutions were prepared in methanol at a concentration of 1 mg/ml and stored in aliquots at -80^o^C. Working stock solutions of each drug were prepared each day from the super stock solutions at a concentration of 100 μg/ml, 10 μg/ml, and 1 μg/ml which were used to spike the calibrators. Food calibrator samples were prepared by spiking food samples at concentrations of 0, 0.0625, 0.125, 0.25, 0.5, 1, 2, 4, 8, and 16 ng/mg. LUT, CRN, and WFA were quantified in mouse food by mixing 100 mg of the calibrator and unknown samples with 1 mL of ethanol (200 proof) and then shaking for 30 minutes. The samples were then centrifuged for 10 minutes at 3,200 g. The supernatant was transferred to 1.5ml microcentrifuge tubes and dried under a stream of warm nitrogen gas. The samples were resuspended in 100 µl of 50/50 mobile phase A/mobile phase B, vortexed, and spun at 17,000 g for 2 minutes. The final extracts were transferred to autosampler vials and 10 µL was injected into the LC/MS/MS for each mode. The peak area ratios for each unknown sample were compared against a linear regression of calibrator peak area ratios to quantify each drug. The concentration of each drug was expressed as ng/mg or ppm of food.

Measurement of Sulindac in Mouse Food using HPLC-UV

Sulindac, sulindac sulfide, sulindac sulfone, and clonazepam were purchased from Sigma-Aldrich Chemical Company (St. Louis, MO). All other reagents were purchased from Thermo Fisher Scientific (Waltham, MA). Milli-Q water was used for the preparation of all solutions. The HPLC system consisted of a Shimadzu SIL 20A HT autosampler, LC-20AD pumps (2), and an SPD-20A UV detector. The LC analytical column was an ACE Excel C18-AR (75 x 3.0 mm, 3 microns) purchased from Mac-Mod Analytical (Chaddsford, PA) and was maintained at 40^o^C during the chromatographic runs using a Shimadzu CT-20A column oven. Mobile phase A contained 50% HPLC grade methanol, pH 3.0 while mobile phase B contained 100% HPLC grade methanol. The flow rate of the mobile phase was 0.5 ml/min. The drugs were eluted with a gradient. The initial mobile phase was 10% B and 1 min. after injection was ramped to 95% B. From 9.0 min. to 12.0 min. the mobile phase was maintained at 95% B. At 12.01 min, it was switched immediately back to 10% B and ran for 3.9 minutes to equilibrate the column before the next injection. All drugs were detected at 340 nm. Clonazepam eluted at 4.25 min, sulindac at 5.35 min, sulindac sulfone at 5.6 min, and sulindac sulfide at 9.2 min.

All drug super stock solutions were prepared in methanol at a concentration of 1 mg/ml and stored in aliquots at -80^o^C. A working stock solution of sulindac and its metabolites was prepared each day from the super stock solutions at concentrations of 100 μg/ml and 10 μg/ml were used to spike the calibrators. Food calibrator samples were prepared by spiking food samples at concentrations of 0, 2.5, 5, 10, and 20 ng/mg. Sulindac was quantified in mouse food by mixing 100 mg of the calibrator and unknown samples with 20 µL of 100 µg/mL clonazepam (internal standard) and 1 mL of acetonitrile then shaking for 30 minutes. The samples were then centrifuged for 10 minutes at 3,200 g. The supernatant was then dried down under warm nitrogen gas. The samples were re-suspended in 100 µl of mobile phase A, vortexed, and spun at 17,000 g for 2 minutes. The final extracts were transferred to autosampler vials and 20 µL was injected into the HPLC/UV. The peak area ratios for each unknown sample were compared against a linear regression of calibrator peak area ratios to quantify sulindac. The concentration of sulindac was expressed as ng/mg or ppm of food.

Measurement of Syringaresinol in Mouse Food Using HPLC-UV

Syringaresinol (SYR) was obtained from Dr. Yousin Suh (Bronx, NY). All other reagents were purchased from Thermo Fisher Scientific (Waltham, MA). HPLC grade methanol was used for the preparation of all solutions. The HPLC system consisted of a Waters 717 autosampler, 515 HPLC pump, and a 2487 Dual wavelength absorbance UV detector. The LC analytical column was an ACE Excel C18-PFP (75 x 3.0 mm, 3 microns) purchased from Mac-Mod Analytical (Chaddsford, PA). The mobile phase was 30% HPLC grade acetonitrile and 70% 20mM KH_2_PO_4_, pH 2.5. The flow rate of the mobile phase was 0.5 ml/min. SYR was detected at 214 nm and eluted at 11 minutes. SYR super stock solution was prepared in methanol at a concentration of 1 mg/ml and stored in aliquots at -80^o^C. A working stock solution of each drug was prepared each day from the super stock solutions at a concentration of 100 μg/ml and used to spike the calibrators. Food calibrator samples were prepared by spiking food samples at SYR concentrations of 0, 37.5, 75, 150, 300, and 600 ng/mg. SYR was quantified in mouse food by mixing 100 mg of the calibrator and unknown samples with 1 mL of mobile phase and then shaking for 30 minutes. The samples were then centrifuged for 10 minutes at 3,200 g. The supernatant was transferred to microcentrifuge tubes and centrifuged at 17,000 g for 2 minutes. The final extracts were transferred to autosampler vials and 20 µL of the final samples were injected into the HPLC/UV. The peak area ratios for each unknown sample were compared against a linear regression of calibrator peak area ratios to quantify SYR. The concentration of SYR was expressed as ng/mg or ppm of food.
